# Motivation and experiences of dentists of primary care dental clusters in Hungary: a qualitative inquiry

**DOI:** 10.3389/froh.2024.1492387

**Published:** 2025-01-13

**Authors:** András Sztrilich, Gergő Túri, Csilla Kaposvári, Rita Teller, István Vingender

**Affiliations:** ^1^ Doctoral School of Health Sciences, Faculty of Health Sciences, Semmelweis University, Budapest, Hungary; ^2^ Epidemiology and Surveillance Centre, Semmelweis University, Budapest, Hungary; ^3^ Doctoral School of Health Sciences, University of Debrecen, Debrecen, Hungary; ^4^ Synthesis Health Research Foundation, Budapest, Hungary; ^5^ Department of Public Health, Semmelweis University, Budapest, Hungary; ^6^ Faculty of Health Sciences, Doctoral School, University of Pécs, Pécs, Hungary; ^7^ Faculty of Humanities, Eötvös Loránd University, Budapest, Hungary

**Keywords:** dental services, cluster practice, group practice, primary care, Hungary, finance, regulation, network

## Abstract

**Background:**

In recent years, dental clusters and networks have been established in primary care in many countries to improve access to services for the population and develop cooperation between providers. In Hungary, the first dental clusters were established in 2021, and currently, one-third of dental practices have already joined a cluster. The study aimed to gather and analyze early experiences regarding the motivation of participation in primary care dental clusters and experiences of implementation.

**Methods:**

Qualitative in-depth individual interviews with primary care dentists (*n* = 21). The study was designed to meet the COREQ criteria for reporting qualitative research. The research team members defined a purposive sample of interviewees. All interviews were conducted from March to April 2024. A qualitative content analysis method was used to analyze the interview transcripts. The WHO health system framework was chosen for the theoretical framework of the analysis.

**Results:**

The motivations for joining a dental cluster were financial reasons, professional development and knowledge exchange. Lack of information and distrust were barriers to joining the dental clusters. Different professional management practices have developed within the clusters. In the interviewees' opinion, the population's access to preventive dental services has not yet changed substantially under the new operational model. The portfolio of services offered by dental clusters could be expanded to include a range of types of care. Digital health technologies and innovative solutions should be developed and widely adopted.

**Conclusions:**

In designing policy measures to promote the broader adoption of the dental cluster model, it is helpful to consider the different factors influencing dentists' decisions during implementation. Dental clusters can benefit the public and dentists, but further development of the model and improvement of the primary conditions for the operation of practices are essential.

## 1 Introduction

The Hungarian health care system has a tax-funded, single-payer health insurance fund that provides health care coverage for nearly all, up to 95% of residents (1, 2). Health services covered by the social health insurance scheme include dental services as well (3). Dental care under the public benefit package is provided in service districts with territorial coverage and has primary and specialised segments (3). Primary care dental services are provided in child, adult or mixed care practices by individual private practices (4). According to the National Health Insurance Fund of Hungary, primary dental and oral health care is responsible for the preventive and curative primary care of dental and oral diseases, while specialised dental care has certain additional competences such as oral surgery, orthodontics, treatment of periodontal disease, paediatric specialist care, dental x-rays. Primary and emergency dental care can be accessed without referral (5).

Some dental care services in Hungary—regardless of age—are free of charge (e.g., emergency care, screening, specialist referral, etc.) check-ups (once a year) and dental and oral diseases related to other underlying conditions, treatment of basic dental and oral diseases, tooth-retaining treatments e.g., dental fillings, dental surgery, removal of dental plaque, treatment of gum lesions) and there are treatments which are subsidized according to the age of the insured person (6). With the aim of strengthening primary health care and its essential role in health promotion, prevention, detection and cure of diseases, primary care dental clusters were established by Government Decree 53/2021 (9.II.) on primary care clusters effective from 14 June 2021 (7). The decree enables primary care dental practices to establish active professional cooperation within the dental clusters, to improve the population's access to preventive dental services and to improve the quality of services by adapting methodological protocols developed by the National Directorate General for Hospitals.

Primary care dental clusters can to be established within a site or in a district, in the vicinity of each other, with at least 5 dental practices without an upper limit. Primary care dental clusters may operate in the following 3 professional and economic forms:

1. The *united district dental cluster* is a close professional and economic collaboration between several primary care dental practices, whereby the participating dental providers give up their independence and jointly merge into health care provider (new legal entity) of primary care dental services.
2. The *integrated district dental cluster* is a close professional and economic cooperation between several primary care dental practices, whereby the participating dental service providers preserve their autonomy and establish a health care provider to perform their tasks in a coordinated manner.
3. The *consortium of district dental cluster* is a professional and economic cooperation between several primary care dental practices, whereby the participating dental service providers enter into a consortium collaboration agreement, while maintaining their legal and financial autonomy, in order to coordinate their service provision tasks and appoint a consortium leader to represent the consortium.

The Decree points out that dental clusters are eligible, upon availability, to apply for EU funds and primary care development grants funded by the National Health Insurance Fund. They can use the funds to upgrade their facilities, employ additional professionals and receive performance payments to support them. They can use the grant to create the conditions for and receive remuneration for the use of additional specialist qualifications, licenses and other skills necessary in the dental practice cluster. Primary care dentists belonging to a practice cluster must provide preventive care for at least 4 h per week, and for each additional 500 patients above 2,000 registered per practice, at least one additional hour per week. From the period of its legislative base until December 31, 2023, 74 primary dental clusters were formed nationwide which represents 30% of dental practices. Up to the date of the qualitative research, primary care dental clusters were set up exclusively in the form of consortium.

In several countries in Europe and North-America, primary care dental clusters and networks have been established in recent years, and their development and expansion have been significantly facilitated by the challenges posed by the COVID-19 pandemic (8–12). As in Hungary, these countries aim to improve access to primary care services, focusing on preventive services (13).

This qualitative research had two main objectives: (1) to identify the motivations for dentists to join or not to join the dental cluster model, and (2) to analyze the initial experiences regarding the implementation of the dental cluster model. The WHO health framework was chosen as the theoretical background to analyze the motivations for joining dental clusters and the functioning of the cluster-based model at a systemic level (14). This framework allowed us to organize our results according to health systems' six main building blocks: financing, physical resources, human resources, governance and leadership, information and research, and service delivery.

## 2 Methods

### 2.1 Research design

The qualitative research study used an in-depth interview study design, conducting interviews with primary care dentists to collect motivations, perspectives and experiences (15). The study was designed to meet the COREQ criteria for reporting qualitative research, provided in Supplementary Appendix 1. All participants provided informed consent prior to participation. Personal information was not collected from participants.

### 2.2 Participants and recruitment

The research team members defined a purposive sample of interviewees. The aim was to interview at least 16 persons and ensure sufficient variation via the following criteria: (a) at least two primary care dentists from every Hungarian region, (b) at least eight dentists from adult practices and eight dentists from mixed practices, (c) at least five dentists from rural area, five dentists from urban area and five from mixed area (urban and rural), (d) at least ten with dental cluster membership and five from solo dentist practice, (e) only one interviewee per dental practice, (f) at least three from every pre-defined age groups. School dentists were not recruited, as they are not falling under the scope of the pertaining legal regulations that established primary care dental clusters.

Primary care dentists were eligible to participate in the interviews if they were members of a dental practice, were willing to provide insights from their involvement and experiences in dental clusters and provided informed e-consent. Possible participants were identified by the research team members using the NHIF online database on primary care providers. Dentists from different regions and practice types were invited via an electronic notice sent to their work email to take part in an individual interview. The willingness to participate could be indicated by filling in a participant information sheet about general sociodemographic information on the dental practice, such as type of practice, geographical region, dental cluster membership, and suitable time for the interview. Participants were contacted via telephone and email to finalize a suitable date and time for the interview, and the informed e-consents were collected by return email before the interviews.

### 2.3 Data collection and processing

The research team has developed a semi-structured interview guide in accordance with the research objectives. Thematic fields were (a) motivations for joining or not joining a dental cluster; (b) experiences in preparing and setting up a dental cluster; (c) experiences in the operation of the dental cluster; (d) opportunities for further development of dental clusters; (e) and population access to preventive dental services. Questions were asked by the Interviewer but not provided to participants. The interview guide was pilot-tested with two dentists.

All interviews were conducted from March to April 2024 by RT, CK and GT. All researchers had previous experience in conducting interviews during qualitative studies evaluating the implementation of healthcare-related interventions. All interviews were recorded using Zoom teleconference. Interviews were transcribed verbatim from the audio by RT, CK, and GT using Microsoft Word, and AS checked transcripts for consistency. Transcripts were not returned to participants for comment. Field notes were made after the interviews by the interviewers. None of the participants knew the Interviewer prior to the interviews. The semi-structured interview guide is provided in Supplementary Appendix 2.

According to the literature on qualitative research methodology, data saturation can be achieved with 6–17 interviews (16, 17). Conceptual saturation occurs in qualitative research when new themes can no longer be identified by conducting new interviews about those identified from existing data. Since it was reasonable to explore and identify a broad range of motivations and experiences among dentists who joined the dental cluster and those who did not, a separate saturation table was used for the two groups. Data saturation was assessed using Guest and colleagues’ methodology with a threshold of 5% for new information (16). Five interviews among dentists who did not join a dental cluster and 13 interviews among dentists who joined a dental cluster were sufficient for thematic saturation.

In the study, a comprehensive data triangulation was carried out based on the methodology of Carter et al., and we found consistent findings from the interviews (18). For investigator triangulation, three experts from different disciplines conducted the interviews, and two experts coded the transcripts and designed the coding system. For theory triangulation, the entire research team was involved to explore the possible interpretations of the data regarding different theories and ultimately selecting the WHO health system framework as the theoretical background for the analysis. The methodological triangulation included interviews, field notes and a collection of relevant legislation, policy documents and literature on the topic. In the data source triangulation, interviews were conducted with dentists of different age groups, genders, practice types, geographical regions and area types.

### 2.4 Analysis

The WHO health system framework was chosen as the theoretical framework for the analysis, which was used to categorize the interview results according to the six main building blocks of health systems (financing, physical resources, human resources, governance and leadership, information and research, and service delivery) (14). This framework has often been used to analyze the functioning of health systems and subsystems (19–21). Participant characteristics are summarized using descriptive statistics, including age, gender, dental practice unit type, dental cluster membership, region, and area type. A qualitative content analysis method was used to analyze the interview transcripts. The analysis was conducted in Atlas.ti 22 software. A coding framework was developed, and each transcript was coded at least twice by CK and SA. CK and SA examined the categories of the coding framework to identify patterns in the data and form new insights. Consensus-building discussions were used to agree upon the themes within the framework, data was integrated for the conclusion during these discussions, and the entire research team took part in interpreting the results.

## 3 Results

### 3.1 Description of participants

In total, 21 primary care dentists participated in the study (Table 1). The audio interviews were an average of 52 min. Participants were from different age groups, slightly more than half were female (11/21, 52%) and the majority worked in mixed dental practices (13/21, 61%). At least two participants were recruited from each region of the country, and the largest proportion of participants had dental practice in an urban area. Majority of interviewees (15/21, 71%) were members of a dental cluster.

**Table 1:** Characteristics of interviewed participants (*n* = 21).

Age groups (years)	30–39	4
	40–49	4
	50–59	6
	60–69	4
	70–79	3
Gender	Female	11
	Male	10
Practice type	Adult	8
	Mixed	13
Cluster member	Yes	15
	No	6
Region	Western transdanubia	2
	Southern transdanubia	3
	Central transdanubia	2
	Central Hungary	4
	Budapest	3
	Southern great plain	2
	Northern great plain	3
	Northern Hungary	2
Area	Urban	9
	Rural	5
	Mixed (rural and urban)	7

Sources: Authors.

The main themes identified in the analysis were grouped according to the blocks of the WHO health systems framework (Table 2). The following section describes these themes in more detail. Identified themes:

**Table 2:** Main findings grouped according to the blocks of the WHO health system framework.

Building block	Themes
Financing	Financial motivations to join a dental cluster were more for human resources.
Physical and Human resources	The early process of implementing primary dental care clusters has not resulted in increased infrastructure or personnel.
Governance and leadership	Different professional management practices have developed within the dental clusters.
	Lack of information and distrust were barriers to joining the dental clusters.
Information and research	Knowledge exchange would benefit dentists participating in clusters and would help some clinics update their clinical protocols
Service delivery	The cluster-based system has not substantially changed preventive service delivery in the first three years of its existence. Population health behaviors were unlikely to be changed in the cluster-based system.

Sources: Authors.

### 3.2 Financing

Financial motivations to join a dental cluster were more for human resources.

Funding for primary dental care has been and continues to be low in Hungary. All dentists reported that the current system does not support the population's dental needs or the participating dentists' business needs. In reforms moving towards performance-based funding, subjects felt that performance limits might actually disincentivize dentists. Dentists would have had to give up a substantial degree of economic autonomy in two of the three types of dental clusters (unified district dental cluster, integrated district dental cluster). The change in ownership status would have transformed them from proprietors to employees, providing more limited options for economic decisions and service portfolio development. In addition, the reimbursement structure would have been more complex and less accessible and transparent to an employee. Overwhelmingly, dentists did not want to give up their economic autonomy in establishing clusters. Therefore, all interviewees chose the third type of dental cluster, the consortium of district dental clusters*,* in which they have maintained their full economic and professional autonomy. Among the financial motivations for joining the dental cluster, human resources factors were the most substantial, allowing interviewees to improve dentists' and assistants' salaries. The additional subsidy for joining a dental cluster allowed dentists to gain an extra source of income. According to the participants, the financial situation of dental practices has improved slightly due to the subsidy.

“If I take out of the amount of funding what I am obliged to pay in wages, the remainder is very much reduced. Almost all the money goes to maintaining the practice, paying regular monthly allowances and contributions.” (ID9)

“The problem with performance-based funding is, for example, the performance volume limit, beyond which it is no longer worth providing more services than a certain volume because it is not reimbursed at full price by the NHIF.” (ID12)

“Anyone who thinks that dental practices can be integrated into a single economic entity does not really know how dentists work. Dentists have been used to operating independently for almost three decades, and I don't think they would support giving up their economic independence.” (ID 2)

“My decision to join the dental cluster was primarily driven by financial considerations. However, the additional subsidy only allowed for an increase in staff wages.” (ID3)

### 3.3 Physical and human resources

The early process of implementing primary dental care clusters has not resulted in increased infrastructure or personnel.

All respondents stated that for the time being, joining a dental cluster has not resulted in sufficient resources for infrastructure and equipment improvements, so these advances are either not made or made later. Since the creation of the dental clusters in 2021, there have been no such tenders to apply jointly for equipment and infrastructure development, but there would be a substantial need for them. The lack of tenders for infrastructure and equipment for dental clusters creates an uneven level of development among primary care providers, as substantial funding was available for such purposes when the GP clusters were established in 2010s. Some respondents perceived that a dental cluster would be a helpful way to reduce the cost of infrastructure and equipment improvements by allowing several practices to jointly purchase a larger quantity of materials and equipment needed for their operations.

“The absence of a subsidy for high-quality dental filling material, which costs 6,000 Forints per gram compared to the standard 2,000 forints, is a significant financial burden. Similarly, the lack of modern dental equipment like drills and CBCT machines adds to the challenges faced by dental practices.” (ID 1)

“During the establishment of the GP clusters, it was possible to improve infrastructure and equipment within the framework of tenders, which is now lacking in the case of the dental clusters, even though the dental profession is inherently more equipment and material-intensive. We joined because we were confident that we could apply as a member of a dental cluster.” (ID 12)

Most participants stated that the shortage of dental assistants and technicians is a severe problem due to an ageing workforce and a lack of replacements. The shortage of dental assistants has been particularly exacerbated by the fact that during the COVID-19 pandemic, doctors' salaries in the health sector increased more than those of assistants, creating dissatisfaction and a lack of motivation among assistants and technicians. However, in the dental cluster model, a pay increase in proportion to the length of time in practice was implemented when the dental cluster was established. During the interview period and from March 2024, a further pay increase was implemented for them in the framework of the additional practice allowance. The majority of interviewees felt that the additional funding for the dental cluster model was only sufficient to pay the existing HR salary increase. Dentists believed it would be helpful to involve additional human resources in the operation of the dental clusters, as is the case in many GP clusters in Hungary. Apart from administrative support jobs, radiographers and dental hygienists were the most frequently mentioned as opportunities for HR expansion.

“In the health sector, medical salaries were increased at the beginning of the COVID-19 pandemic, but there is still a serious gap between the salaries of doctors and assistants. I have noticed that finding a suitable assistant who will come to work for that kind of money is getting harder and harder.” (ID 8)

“The dental cluster model has not changed much the situation of an increasing shortage of professional staff.” (ID 19)

### 3.4 Governance and leadership

Lack of information and distrust were barriers to joining the dental clusters.

Lack of information, fear, and general distrust were barriers for dentists who did not join a dental cluster. Some dentists highlighted the lack of transparency of the dental cluster model's aims and conditions of application. In contrast, others highlighted the increasing administrative burden and the risk of unilateral changes to the operating conditions. The possibility of compulsory or underpaid overtime for dentists working in the dental cluster or compulsory substituting dental practices in remote municipalities was considered a risk. Therefore, joining the dental cluster was hindered by management processes that were sometimes responsible for a lack of transparency and unilateral regulations, which, according to the respondents, have been the case in several examples in recent decades.

“I was intimidated by the lack of transparency and information, so I didn't join the dental cluster. I was afraid that if I joined, I would later be obliged to do things I didn't want to do.” (ID 18)

“I preferred to stay away from the dental clusters. Partly because of the administrative burden, and partly because I was afraid that the dental cluster might threaten this kind of greater control and independence of the practices.” (ID 17)

“I did not join because I thought it was a real risk that after joining, I would be obliged to carry out activities that were not included in the tasks when the call for applications was launched.” (ID 20)

Different professional management practices have developed within the dental clusters.

The dentists who joined a dental cluster reported different professional management practices. According to some of the respondents, the consortium-led practice is more passive. It does not perform any management tasks other than organizing administrative tasks. In contrast, other dentists have experienced the consortium-led practice of actively organizing professional meetings and internal training. These discussions and internal training sessions were perceived particularly valuable for younger dentists beginning their careers. The different types of professional management were more likely to be due to managers' different personal motivations and commitments. Both active and passive management styles were observed in clusters operating in urban and rural areas and with older and relatively young managers. The intensity of cooperation between dentists and general practitioners and the need for closer collaboration also varied.

“Within the dental cluster, there is active cooperation between practices; we hold regular meetings, and every three months, we organize face-to-face meetings and internal professional training.” (ID 12)

“There have been a few meetings in the dental cluster in the last six months, but these have not been long, rather technical and administrative. There has been no professional guidance from the head of the dental cluster or the national level.” (ID 21)

“There would be benefits from closer cooperation between dental and general practices. For dental treatment, the GP could provide useful information on the patient’s medical history and chronic diseases affecting the treatment or medication. Many GPs are open to this and can cooperate with them. It is more the older GP colleagues who are reluctant.” (ID 6)

“I do not see much point in closer, formalized GP collaboration. We can usually get the information we need for patient care from GPs.” (ID13)

Some dentists felt that there had been no significant change in professional management at national level since the transition to the dental cluster model. Other interviewees argued that the situation and functioning of primary dental care is less predictable due to policy interventions with different objectives and directions. Some dentists stressed that they considered the different approaches to financing unfair regarding the remuneration of doctors working in different health subsystems.

“Any system can only work well if it has a solid foundation and a predictable future. The funding and conditions for primary care dentistry have constantly changed and become hectic in recent years. If funding were to become more predictable, then a set of requirements could be developed in parallel and should be held to account.” (ID 12)

“In the health sector, doctors’ salaries have been increased substantially, but this increase has not been linked to performance in many places, such as hospitals and specialised care. Therefore, doctors receive increased pay in many places even if they underperform compared to their previous performance. By contrast, in dental practice, funding is linked to performance from 2023.” (ID 6)

### 3.5 Information and research

Knowledge exchange would benefit dentists participating in clusters and would help some clinics update their clinical protocols.

In addition to funding, the other main motivating factor for joining a dental cluster was knowledge exchange. Dental clusters can facilitate the exchange of knowledge and information between dental practices, thus contributing to the development of dentists' and dental assistants' knowledge and practices and exchanging good practices. This motivational factor was particularly prevalent among dentists in the younger age group. However, subjects in all age groups considered knowledge exchange's importance and benefits essential.

“Besides joining a dental cluster, I also had a professional motivation, because I thought that if several specialists set up a dental cluster, it would be possible to take care of more complicated cases or discuss them, from which we could learn together, discuss professional issues together.” (ID 19)

Dentists also expressed a strong need to provide free professional training and to maintain and update primary care guidelines and protocols. Most dentists felt it would be helpful to continuously maintain and update primary care dentistry guidelines and support and monitor their translation into daily practice. Some respondents stated a need to provide free professional training at a location geographically close to the area of operation of the dental cluster. A related area also discussed was digital technologies as a potential tool to support knowledge exchange, training and the provision of innovative services. Online professional platforms facilitate access to knowledge and knowledge exchange between dentists. Training courses delivered online can save significant time and costs compared to on-site training. In addition, a number of health informatics and digital technologies already exist that allow for more efficient administration and diagnostic procedures and treatments, and their broader adoption in dental clusters would be desirable.

“The work of the dental cluster would be enhanced if there were applicable and adaptable professional guidelines and protocols. It would also be useful to provide free continuing professional development courses on various topics, such as managing malodor in the practice or resolving legal disputes between dentists and patients.” (ID 15)

“Health information systems could better help the dental clusters with their administrative burden. For example, whenever there was a need, the accessibility and retrieval of existing data with EESZT were often problematic. Many good things in health informatics are not yet exploited, such as a shared appointment booking system or sharing x-ray images between practices. Patient education could also be done using online platforms and electronic solutions.” (ID 6)

### 3.6 Service delivery

The cluster-based system has not substantially changed preventive service delivery in the first three years of its existence. Population health behaviors were unlikely to be changed in the cluster-based system.

According to most participants, access to dental care for the general public in the dental clusters has mostly stayed the same. Although a condition of joining was that certain preventive services had to be provided, most dentists stated they provided them to the same extent before joining. The dentists interviewed thought the mandatory preventive surveys and questionnaires in the dental cluster model are useful, but patients' health behavior remains unchanged. The demand for preventive services is low, and patients mostly visit the dentist's office only with an established dental problem. Demand for primary care dental services and prevention is affected by a lack of knowledge and/or misconceptions among the population about these services. In order to improve the population's demand for primary care services, a broad health education on dental care would be needed.

“I haven't noticed any difference in the population’s access to preventive services since I became a member of a dental cluster. I provided preventive services and patient information before establishing the dental cluster.” (ID 13)

“The problem, I think, is that the population is seeking preventive services at a shallow level. I'd love to do more preventive care and counselling, but people often come to us when they have a problem, such as toothache. So it would be important to educate patients, raise their awareness, and motivate them to attend regular dental check-ups even if they don't have a problem.” (ID 4)

However, the number of preventive dental services can only be increased to a limited extent due to a lack of capacity on the provider side. According to some dentists, there is limited scope for increasing the number of preventive dental services, which may be due to systemic, funding, organizational and human resource problems. Based on the respondent's perception, the systemic problem hindering capacity development cannot be effectively resolved if health policy only addresses some of the underlying factors. For dental clusters to contribute to solving capacity problems by recruiting new specialists, it would be necessary to change existing funding methods and professional protocols, which currently do not entitle them to capacity development.

“At the moment, we should allocate 15 min per patient. However, the administrative burden in dental care is significant. When a patient comes in, I do a thorough assessment, an oncological screening, a bleeding index measurement, and oral care advice. If dental treatment is also required, that’s more than half an hour with administration. Even for a dental hygienist who does his job well, it is impossible to care for a patient in 15 min.” (ID 3)

“The alpha and omega of everything is funding. Until the funding conditions are in place to enable dental clusters to provide a higher level of service to those treated in primary care, this will not happen. A dentist with an endodontic specialty exam cannot report a higher level of care codes, only primary care dental codes if they are in a primary care practice. The only way to include different specialities in primary care is to assign funding to them. So you can only play small football with a small amount of money. Right?” (ID 12)

Regarding primary care service development, the portfolio of services offered by dental clusters could be expanded to include a range of types of care, such as oral surgery and orthodontics, but this would require changes in financing methodologies. Another challenge is how to account for the cost of materials and equipment for dentists and health professionals jointly employed in a dental cluster, as this is very different from the independent operating practice that has been established so far. At the same time, the win-win nature of this operation model and the development potentials of introducing primary care dental clusters have also been emphasized by some interviewees.

“It could also be that dentists working within a dental cluster might employ specialists together and jointly pay for a surgeon, orthodontist, or periodontist so that a specialist could complement or help us. But the implementation would not be easy because then it would have to be agreed whose practice these jointly employed people would work in, whose equipment and materials they would use.” (ID 4)

“In dentistry, it would be an added value for the population if we had more doctors with a dental specialisation, such as an oral surgeon, a periodontist, an orthodontist, or if we could install an imaging diagnostic unit and then everyone could send their patients there, and it would really be within reach, and then we could achieve a positive change in operations. (ID 9)”

## 4 Discussion

Our study is the first in Hungary to assess the motivations for joining dental clusters. According to participants in this research, the motivations for joining a dental cluster were financial reasons, professional development and knowledge exchange. Lack of information and distrust were barriers to joining the dental clusters. These findings suggest that in designing policy measures to promote the broader adoption of the dental cluster model, it is helpful to consider the different factors influencing dentists' decisions and experiences.

Several studies have shown that financial incentives can support the development of collaborations, and Hungarian dental clusters can serve as an example of this practice (22, 23). The Dutch and English examples show that the role of dental communities of practice is also essential in knowledge exchange and further training of professionals, which already exists in some of the Hungarian dental clusters (8–10). However, international experience also shows that unequal power relations in collaborations and clusters are less conducive to developing effective primary care collaborations (22, 24). Our results align with research in other areas of the Hungarian health sector, where the lack of transparency and trust in the system has been identified as a problem (25).

In addition, our study was the first to evaluate the experiences of the first three years of implementing dental clusters in Hungary based on interviews with dentists. According to participant in this research, the cluster-based system has not substantially changed preventive service delivery in the first three years of its existence.

Our results are in line with the findings from prior research indicating that changing from a single-practitioner model to an interdisciplinary team-based model has many challenges and takes time. In the US for example, practice consolidation in dentistry continues to develop towards having fewer dentists in solo practice and more in groups of various configurations and characteristics, and having more employees than practice owners (26). Studies from Canada about the experiences of introducing the interprofessional team-based practice model called family health teams showed that primary care teamwork requires a learning and change of organizational work culture in addition to being physically close and having a mandate to work together (27, 28). Our findings also reflect the experience of international research that various factors and preferences affect the decision about choosing certain practice types (29). Demographics, specialization, career stage, perceived autonomy on business and professional decisions, and the location (state) are associated with the preference of practice type choices. Compared to the Hungarian dental cluster model, a broader, active collaboration between primary care dentists, general practitioners, pharmacies, and dentists working in specialized care has been established in the Netherlands and the United Kingdom (9, 10, 30). The possibility of broader collaboration has also been identified as an opportunity for improvement by some dentists in our study, thus in line with international trends.

Two recent international systematic reviews have examined the experiences and good practices of primary care provider collaborations and clusters in European countries, North America and Australia (22, 24). Both studies found that for collaboration to be effective, the same principles and goals must guide the collaborating actors. Also, essential factors for successful collaboration are continuous communication, a well-defined division of tasks, and knowledge of each other's professional responsibilities. Seaton et al.'s analysis also highlighted that cooperation can also be facilitated if the different primary care actors are spatially close to each other, which is in line with the opinion of some of the dentists in our study.

Our research has the following limitations. Firstly, we assessed the first three years of the development and operation of dental clusters, i.e., the initial experience when the health system was affected by several external factors, including the COVID-19 pandemic, the economic crisis and inflation. However, the experience of this initial period can also provide helpful information for the further development of the dental cluster model. Secondly, the financing of primary care dentistry has changed significantly on two occasions in the last three years, which may have affected different dental practices in different areas in different ways. Thus, dentists may have evaluated the primary care system in different ways. This may also have influenced their motivations for joining or not joining a dental cluster, of which several examples are described in our research. Thirdly, subjectivity is a characteristic of qualitative research, thus the findings may not be generalizable. However, our study included participants with different viewpoints and factors, and the analysis was conducted by professional standards and with the involvement of the whole research team (15).

A future area of research is to identify how cooperation can be developed between dental clusters and several other primary care actors, such as GPs and Health Promotion Offices (31–35). Based on the results of our research and other national research, there is a strong demand from primary care actors in Hungary for the development and adaptation of digital health technologies, and it would be helpful to explore the potential of this area in more detail (36–38). Domestic and international studies have shown that the COVID-19 pandemic has had a significant impact on health system actors worldwide and has negatively affected the population's access to health services (21, 39, 40). It would be helpful to investigate in more detail the impact of the pandemic on the functioning of Hungarian primary dental care to enable the system to respond more quickly and effectively to similar challenges in future health emergencies. In addition, it would be useful to evaluate the mid and long-term experience and outcomes of the operation of dental clusters using qualitative and quantitative methods.

## 5 Conclusion

This qualitative study found that primary care dentists' motivations for joining a dental cluster in Hungary were mainly financial reasons, professional development and knowledge exchange. Among barriers to joining primary care dental clusters, lack of information and distrust were mentioned by interviewees. Different professional management practices have developed within the clusters. Many interviewees suggested that the portfolio of services offered by dental clusters could be expanded to include a range of types of care and digital health technologies and innovative solutions should be developed and widely adopted. The interviewed dentists in different part of Hungary have not yet noticed a substantial change in the access of the population to preventive dental services under the new operational model. These findings suggest that in designing policy measures to promote the broader adoption of the dental cluster model, it is helpful to consider the different factors influencing dentists’ decisions and experiences. In conclusion, it is viewed that primary dental clusters can benefit both the public and the dentists, but further development of the model and improvement of the primary conditions for the operation of clusters are essential.

## Data Availability

The original contributions presented in the study are included in the article/Supplementary Material, further inquiries can be directed to the corresponding author.

## Supplementary material

Supplementary Appendix 1 Consolidated criteria for reporting qualitative research (COREQ) Checklist

Supplementary Appendix 2 Interview Guide.

Supplementary Appendix 3 Characteristics of interview partners.
