# Supplementary material for: Motivation and experiences of dentists of primary care dental clusters in Hungary: a qualitative inquiry
Source: Front Oral Health. 2025 Jan 13;5:1492387. doi: 10.3389/froh.2024.1492387 (PMC11770032; doi:10.3389/froh.2024.1492387)
Supplement: Supplementary Appendix 1 — Consolidated criteria for reporting qualitative research (COREQ) Checklist [file Table1.docx]

Appendix 1. Consolidated criteria for reporting qualitative research (COREQ) Checklist. Experiences of primary care dental group practices in Hungary – a qualitative inquiry

| **Domain 1: Research team and reflexivity** |  |
| --- | --- |
| *Personal Characteristics* |  |
| 1. Interviewer/facilitator: Which author/s conducted the interview or focus group? | Interviews were conducted by RT, or CK, or GT. |
| 2. Credentials: What were the researcher’s credentials? E.g. PhD, MD | RT holds a BA in Sociology and an MSc in Economics, CK holds an MSc in Epidemiology and an MA in Psychology, GT holds a BA in Sociology and an MSc in Health Economics. |
| 3. Occupation: What was their occupation at the time of the study? | RT is a Researcher, CK is an Epidemiologist, GT is an Epidemiologist and Health Economist. |
| 4. Gender: Was the researcher male or female? | RT and CK both identify as female, GT identify as male. |
| *Experience and training* |  |
| 5. What experience or training did the researcher have? | RT has conducted  >10 individual structured interviews with research participants.  CK has organized, supervised and taken notes for >20 focus groups and led 10 focus groups and conducted  > 50 structured interviews with research participants.  GT has organized, supervised and taken notes for  >10 focus groups, led 3 focus groups and conducted  >30 individual semi-structured interviews with research participants. |
| *Relationship with participants* |  |
| 6. Relationship established: Was a relationship established prior to study commencement? | RT, CK and GT had no prior relationships with any of the participants who took part. |
| 7. Participant knowledge of the interviewer: What did the participants know about the researcher? e.g. personal goals, reasons for doing the research | None of the participants knew the interviewer prior to the interviews. All participants read and signed the consent form, so they knew the reasons and goals for conducting the interview. |
| 8. Interviewer characteristics: What characteristics were reported about the interviewer/facilitator? e.g. Bias,  assumptions, reasons and interests in the research topic | No characteristics were reported. |
| **Domain 2: study design** |  |
| *Theoretical framework* |  |
| 9. Methodological orientation and Theory: What methodological orientation was stated to underpin the study? e.g. grounded theory,  discourse analysis, ethnography, phenomenology, content analysis | Qualitative content analysis. |
| *Participant selection* |  |
| 10. Sampling: How were participants selected?  e.g. purposive, convenience, consecutive, snowball | Purposive |
| 11. Method of approach: How were participants approached? e.g. face-to-face, telephone, mail, email | Participants were invited via email. |
| 12. Sample size: How many participants were in the study? | 45 participants were invited 21 interviews were conducted |
| 13. Non-participation How many people refused to participate or dropped out? Reasons? | 24 dentist did not respond to the invitation. |
| *Setting* |  |
| 14. Setting of data collection: Where was the data collected? e.g. home, clinic, workplace | Online using Zoom teleconference |
| 15. Presence of non-participants: Was anyone else present besides the participants and researchers? | No |
| 16. Description of sample What are the important characteristics of the sample? e.g. demographic data, date Data collection | Demographic data |
| 17. Interview guide: Were questions, prompts, guides provided by the authors? Was it pilot tested? | Questions were asked by the Interviewer but not provided to participants. Interview guide was pilot tested with two dentists. |
| 18. Repeat interviews: Were repeat interviews carried out? If yes, how many? | No. |
| 19. Audio/visual recording: Did the research use audio or visual recording to collect the data? | Audio recording was used to collect data via Zoom teleconference. |
| 20. Field notes: Were field notes made during and/or after the interview or focus group? | Yes, after the interviews. |
| 21. Duration: What was the duration of the interviews or focus group? | 45 minutes |
| 22. Data saturation: Was data saturation discussed? | Yes |
| 23. Transcripts returned: Were transcripts returned to participants for comment and/or correction? | No |
| **Domain 3: analysis and findings** |  |
| *Data analysis* |  |
| 24. Number of data coders: How many data coders coded the data? | Two study members coded the data with discrepancies checked by third study member. |
| 25. Description of the coding tree: Did authors provide a description of the coding tree? | Yes |
| 26. Derivation of themes: Were themes identified in advance or derived from the data? | Yes, themes derived from the data. |
| 27. Software: What software, if applicable, was used to manage the data? | All interviews were transcribed verbatim from the audio by RT, CK and GT in Microsoft Word, thematic analysis produced with Atlas.ti 22 sotftware. |
| 28. Participant checking: Did participants provide feedback on the findings? | No |
| *Reporting* |  |
| 29. Quotations presented: Were participant quotations presented to illustrate the themes  / findings? Was each quotation identified?  e.g. participant number | Yes |
| 30. Data and findings consistent: Was there consistency between the data presented and the findings? | Yes |
| 31. Clarity of major themes: Were major themes clearly presented in the findings? | Yes |
| 32. Clarity of minor themes: Is there a description of diverse cases or discussion of minor themes? | Yes |
