# Supplementary material for: Motivation and experiences of dentists of primary care dental clusters in Hungary: a qualitative inquiry
Source: Front Oral Health. 2025 Jan 13;5:1492387. doi: 10.3389/froh.2024.1492387 (PMC11770032; doi:10.3389/froh.2024.1492387)
Supplement: Supplementary Appendix 2 — Interview Guide. [file Table2.docx]

Appendix 2. Interview Guide. Experiences of primary care dental group practices in Hungary – a qualitative inquiry

The interview guide has been translated from Hungarian.

**Introduction of the researcher**

**Brief information about the study**

You have been invited to participate in an online interview for the study on the experiences of dental clusters' development and functioning. An independent researcher from Semmelweis University will conduct the interview.

**Main purpose of the research**

We would like to get a comprehensive picture regarding the following themes:

- What are your experiences in developing dental clusters?

- What factors have influenced you to join a dental cluster or to decide not to participate?

- How has the availability of primary care dental services, especially preventive care, changed with the creation of dental clusters?

**Technical information**

Participation in the interview is entirely voluntary. You are under no obligation to answer all questions or to participate in the interview. Your decision to not answer a question or not to participate will be fully respected and will not result in any form of discrimination.

The interview, which will take 45-60 minutes, will be conducted with utmost confidentiality. The entire interview will be audio-recorded, but no one will be identified.

**Interview questions**

**Motivations for joining or not joining a dental cluster**

- Why did you decide to join or not to join a dental cluster?

**Experiences in preparing and setting up a dental cluster**

- What was your experience in preparing and setting up the dental cluster?

- What supporting and/or hindering factors have you experienced?

- What measures and circumstances helped or hindered the formation?

**Experiences in the operation of the dental cluster**

- In your opinion, how have the conditions for the operation of the dental practice changed in the following areas:

o financing,

o human resources,

o human resources. o equipment,

o professional management,

o professional cooperation

o funding/development opportunities.

**Opportunities for further development of dental clusters**

- In your opinion, which areas could be further developed in the functioning of dental clusters?

- How could the creation of further dental clusters be promoted?

**Access of the population to preventive services**

- How has the population's access to preventive dental services changed since the creation of the dental clusters?

**Concluding the interview**

- Are there any thoughts that have not been expressed but that you think are important to share on this topic?
