# Supplementary material for: Motivation and experiences of dentists of primary care dental clusters in Hungary: a qualitative inquiry
Source: Front Oral Health. 2025 Jan 13;5:1492387. doi: 10.3389/froh.2024.1492387 (PMC11770032; doi:10.3389/froh.2024.1492387)
Supplement: Supplementary Appendix 3 — Characteristics of interview partners. [file Table3.docx]

Appendix 3. Characteristics of interview partners. Experiences of primary care dental group practices in Hungary – a qualitative inquiry

| **ID** | **Age** | **Gender** | **Unit type** | **Cluster member** | **Region** | **Area** | **Duration of interview (hh:mm)** |
| --- | --- | --- | --- | --- | --- | --- | --- |
| 1 | 60-69 | female | Mixed | Yes | Central Hungary | Mixed | 00:56 |
| 2 | 50-59 | female | Mixed | Yes | Northern Hungary | Urban | 00:45 |
| 3 | 70-79 | male | Adult | Yes | Northern Great Plain | Mixed | 01:18 |
| 4 | 50-59 | female | Mixed | Yes | Central Hungary | Mixed | 00:49 |
| 5 | 70-79 | male | Adult | Yes | Budapest | Urban | 00:46 |
| 6 | 40-49 | male | Mixed | Yes | Central Transdanubia | Rural | 00:51 |
| 7 | 30-39 | female | Mixed | Yes | Southern Great Plain | Mixed | 00:59 |
| 8 | 40-49 | female | Adult | No | Budapest | Urban | 0:42 |
| 9 | 50-59 | female | Mixed | Yes | Central Hungary | Rural | 0:49 |
| 10 | 60-69 | male | Mixed | Yes | Southern Great Plain | Rural | 0:57 |
| 11 | 40-49 | female | Adult | Yes | Central Hungary | Urban | 0:55 |
| 12 | 50-59 | male | Mixed | Yes | Budapest | Urban | 1:10 |
| 13 | 60-69 | male | Adult | Yes | Central Hungary | Urban | 0:59 |
| 14 | 50-59 | male | Adult | No | Western Transdanubia | Mixed | 0:39 |
| 15 | 30-39 | female | Mixed | Yes | Southern Transdanubia | Rural | 1:07 |
| 16 | 70-79 | female | Adult | No | Northern Great Plain | Urban | 00:48 |
| 17 | 60-69 | female | Mixed | No | Northern Hungary | Mixed | 00:41 |
| 18 | 50-59 | male | Adult | No | Southern Transdanubia | Urban | 00:45 |
| 19 | 30-39 | female | Mixed | Yes | Southern Transdanubia | Urban | 0:59 |
| 20 | 40-49 | male | Mixed | No | Northern Great Plain | Mixed | 00:36 |
| 21 | 30-39 | male | Mixed | Yes | Western Transdanubia | Rural | 00:47 |
